# Trans‐Ethnic Fine‐Mapping of the Major Histocompatibility Complex Region Linked to Parkinson's Disease

**DOI:** 10.1002/mds.28583

**Published:** 2021-05-11

**Authors:** Tatsuhiko Naito, Wataru Satake, Kotaro Ogawa, Ken Suzuki, Jun Hirata, Jia Nee Foo, Eng‐King Tan, Tatsushi Toda, Yukinori Okada

**Affiliations:** ^1^ Department of Statistical Genetics Osaka University Graduate School of Medicine Suita Japan; ^2^ Department of Neurology, Graduate School of Medicine The University of Tokyo Tokyo Japan; ^3^ Department of Neurology Osaka University Graduate School of Medicine Suita Japan; ^4^ Pharmaceutical Discovery Research Laboratories Teijin Pharma Limited Hino Japan; ^5^ Lee Kong Chian School of Medicine Nanyang Technological University Singapore Singapore Singapore; ^6^ Human Genetics, Genome Institute of Singapore, A*STAR Singapore Singapore; ^7^ Department of Neurology, National Neuroscience Institute Singapore General Hospital Singapore Singapore; ^8^ Duke‐National University of Singapore Medical School Singapore Singapore; ^9^ Laboratory of Statistical Immunology, Immunology Frontier Research Center Osaka University Suita Japan; ^10^ Integrated Frontier Research for Medical Science Division Institute for Open and Transdisciplinary Research Initiatives, Osaka University Suita Japan

**Keywords:** Parkinson's disease; HLA; MHC; fine‐mapping; trans‐ethnic analysis

## Abstract

**Background:**

Despite evidence for the role of human leukocyte antigen (HLA) in the genetic predisposition to Parkinson's disease (PD), the complex haplotype structure and highly polymorphic feature of the major histocompatibility complex (MHC) region has hampered a unified insight on the genetic risk of PD. In addition, a majority of the reports focused on Europeans, lacking evidence on other populations.

**Objectives:**

The aim of this study is to elucidate the genetic features of the MHC region associated with PD risk in trans‐ethnic cohorts.

**Methods:**

We conducted trans‐ethnic fine‐mapping of the MHC region for European populations (14,650 cases and 1,288,625 controls) and East Asian populations (7712 cases and 27,372 controls). We adopted a hybrid fine‐mapping approach including both HLA genotype imputation of genome‐wide association study (GWAS) data and direct imputation of HLA variant risk from the GWAS summary statistics.

**Results:**

Through trans‐ethnic MHC fine‐mapping, we identified the strongest associations at amino acid position 13 of HLA‐DRβ1 (*P* = 6.0 × 10^−15^), which explains the majority of the risk in *HLA‐DRB1*. In silico prediction revealed that *HLA‐DRB1* alleles with histidine at amino acid position 13 (His13) had significantly weaker binding affinity to an α‐synuclein epitope than other alleles (*P* = 9.6 × 10^−4^). Stepwise conditional analysis suggested additional independent associations at Ala69 in HLA‐B (*P* = 1.0 × 10^−7^). A subanalysis in Europeans suggested additional independent associations at non‐HLA genes in the class III MHC region (*EHMT2*; *P* = 2.5 × 10^−7^).

**Conclusions:**

Our study highlights the shared and distinct genetic features of the MHC region in patients with PD across ethnicities. © 2021 The Authors. *Movement Disorders* published by Wiley Periodicals LLC on behalf of International Parkinson and Movement Disorder Society

Parkinson's disease (PD; Online Mendelian Inheritance in Man: 168600) is one of the most common neurodegenerative diseases with its characteristic motor symptoms described as parkinsonism and nonmotor symptoms. The core pathology is the progressive loss of dopaminergic neurons in the substantia nigra with deposition of protein aggregates containing α‐synuclein.^1^ Aberrant functioning of the immune system in the nervous system has been proposed as a critical contributor to the pathogenesis of PD.^2^ Consistently, some of genetic loci identified by genome‐wide association studies (GWAS) are related to immune function.^3^, ^4^, ^5^, ^6^ Among them, the human leukocyte antigen (HLA) genes in the major histocompatibility complex (MHC; 6p21.3) have been implicated in the pathogenesis of PD.^7^, ^8^ For example, HLA molecules bind to α‐synuclein and trigger T cell activation, and its reactivities might depend on the variations in HLA alleles.^7^ Thus, the elucidation of the risk‐associated HLA variants should contribute to further understanding of its pathogenesis.

The association of a variant in the HLA region with PD was first reported in 2010, which was a noncoding variant influencing the expression of HLA‐DR and HLA‐DQ.^9^ Since then, different studies have reported the associations in various aspects, including haplotypes and regulatory variants,^10^ single nucleotide polymorphisms (SNPs) along with smoking history,^10^ and specific combinations of amino acid polymorphisms in *HLA‐DRB1*.^11^ Although they contributed to the elucidation of etiological involvement of HLA on PD, there lacks unified insight on risk‐associated genetic factors derived from a comprehensive MHC region‐wide investigation using large cohorts. In addition, the majority of the reports have focused on European populations, and the evidences for other populations are scarce.^12^, ^13^ The reason behind these should be not only sample size but also complex sequence variations and population‐specific linkage disequilibrium (LD) structures of the MHC region that differ substantially among ethnicities.^14^, ^15^

HLA allelic imputation has successfully contributed to the fine‐mapping of causal risk variants of human complex traits within the MHC region.^16^, ^17^, ^18^, ^19^ By using a population‐specific reference panel, it has achieved high imputation accuracies not only for classical HLA alleles but also for amino acid polymorphisms.^20^ Furthermore, MHC fine‐mapping for a trans‐ethnic cohort can boost its power of revealing genetic features that affect complex diseases beyond ethnicities by removing confounding by linkage.^21^, ^22^ We performed trans‐ethnic MHC fine‐mapping of PD using GWAS data in European and East Asian populations and identified that specific amino acid positions of HLA‐DRβ1 and HLA‐B were independently associated with PD risk across ethnicities. Furthermore, the risk‐associated alleles of *HLA‐DRB1* presented variable binding affinity to a known α‐synuclein epitope in in silico prediction, suggesting their functional role to the pathogenesis of PD.

## 1 Methods

### 1.1 Study Design

**FIG. 1:**
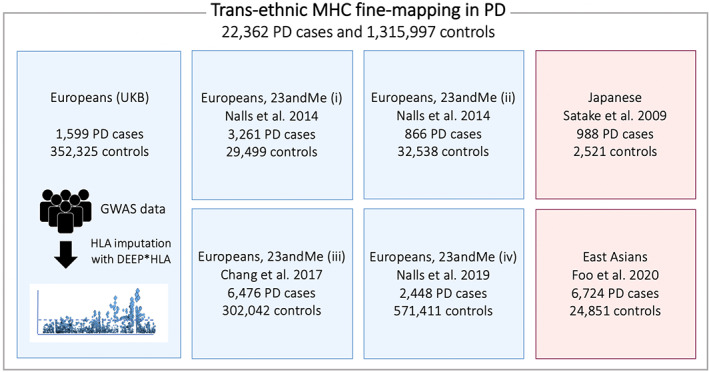
The participants and workflow of our study. We conducted the trans‐ethnic human leukocyte antigen (HLA) fine‐mapping of the Parkinson's disease (PD) risk. Our study included in total seven PD genome‐wide association studies (GWAS) from European and east Asian populations (22,362 PD cases and 1,315,997 controls). UKB, UK Biobank. [Color figure can be viewed at wileyonlinelibrary.com]

A summary of the participants and workflow of our study is shown in Figure 1. First, we applied HLA imputation to individual GWAS genotype data of the UK Biobank (UKB) cohort and conducted association analysis of HLA variants with PD risk (1599 PD cases and 352,325 controls).^23^, ^24^ Then we conducted direct imputation of PD risk statistics of the HLA variants from the PD GWAS summary statistics of European populations obtained from 23andMe, Inc. (14,650 PD cases and 1,288,625 controls; *n*_Study_ = 4)^4^, ^6^, ^25^ and those of East Asian populations (7712 PD cases and 27,372 controls; *n*_Study_ = 2 from Japanese and other East Asians).^5^, ^26^ Finally, we performed trans‐ethnic MHC fine‐mapping using in total seven GWAS summary statistics of European and East Asian populations (22,362 PD cases and 1,315,997 controls; *n*_Study_ = 7).

### 1.2 Participants of UKB

The UKB comprises health‐related information and genotype data of approximately 500,000 individuals who were recruited from across the United Kingdom in 2006 to 2010.^23^ Among them, we used GWAS data from 1599 PD cases and 352,325 controls of British genetic ancestry enrolled in UKB. The detailed sample and genotype quality control (QC) process are described elsewhere.^22^, ^24^ In the current study, we selected individuals who had a history of a diagnosis of PD in hospital records (*International Classification of Diseases, Tenth Revision* code G20 in UKB data‐field code of 41,202 and 41,204) as the PD cases. The controls were selected as individuals who did not have histories of PD in the hospital records or in the self‐reported diagnoses (coding of 1262 in UKB data‐field code of 20,002). In addition, we excluded individuals who had records of autoimmune diseases or some infectious diseases and malignant tumors that have clear associations in the MHC region^19^, ^27^ from the controls (the full lists of excluded diseases are shown in Tables S1 and S2).^16^

### 1.3 Fine‐Mapping of the MHC Region on UKB PD GWAS Data

We defined the HLA variants as single nucleotide variants (SNVs) in the MHC region and classical two‐digit and four‐digit biallelic HLA alleles of class I and II HLA genes (*HLA‐A*, *HLA‐C*, *HLA‐B*, *HLA‐DRB1*, *HLA‐DQA1*, *HLA‐DQB1*, *HLA‐DPA1*, and *HLA‐DPB1*) along with biallelic HLA amino acid polymorphisms corresponding to the respective residues and multiallelic HLA amino acid polymorphisms for each amino acid position. We performed the MHC fine‐mapping in the same process as our previous study.^16^ We prephased the GWAS data with Eagle (version 2.3). Then we applied DEEP*HLA, a multitask convolutional deep‐learning method for HLA allelic imputation, to determine classical two‐digit and four‐digit biallelic HLA alleles.^22^ We used the Type 1 Diabetes Genetics Consortium (T1DGC) reference panel as an imputation reference panel (*n* = 5225). The dosages of biallelic HLA amino acid polymorphisms were determined according to the imputed four‐digit classical allele dosages. As postimputation filtering, we removed the biallelic alleles of which the *r* ^*2*^ in 10‐fold cross‐validation were lower than 0.7. The SNVs in the MHC region were imputed using minimac3 (version 2.0.1). We applied stringent postimputation QC filtering of the variants (minor allele frequency ≥ 0.01 and imputation score *r* ^*2*^ ≥ 0.7).

We evaluated the associations of the HLA variants with risk of PD using a logistic regression model assuming additive effects of allele dosages on a log‐odds scale as described elsewhere.^19^, ^22^ To robustly account for potential population stratification, we included the top 10 principal components (PCs) obtained from the GWAS genotype data (not including the MHC region) as covariates in the regression model. The PCs were calculated using the smartpca program of EIGENSTRAT with default settings.^28^ We also included age, sex, ascertainment center, and genotyping chip of individuals as covariates. To conduct a conditional analysis, we included a target variant to the regression model as a covariate. When conditioning on a specific amino acid polymorphism, we included all the alleles in the target position.

### 1.4 PD GWAS Summary Statistics Used in Our Study

For the European data, we obtained the PD GWAS summary statistics within the MHC region deposited in 23andMe, including the studies of Nalls et al in 2014 (866 PD cases and 32,538 controls, and 3261 cases and 29,499 controls, separated by the genotype platforms),^25^ Chang et al (6476 PD cases and 302,042 controls),^4^ and post‐Chang (2448 PD cases and 571,411 controls).^6^ We did not use a publicly available result of meta‐analysis conducted by Nalls et al^6^ in 2019 considering the sample overlaps and heterogeneity in analytical methods (it includes a result of GWAS‐by‐proxy for the UKB individuals). For the East Asian data, we used the PD GWAS summary statistics of the Japanese population (988 PD cases and 2521 controls)^26^ and the GWAS meta‐analysis result of the East Asian population (6724 PD cases and 24,851 controls).^29^

### 1.5 Fine‐Mapping of the MHC Region on PD GWAS Summary Statistics

The MHC fine‐mapping from the GWAS summary statistics was performed based on the inference from the approximation of *z* scores to multivariate normal distribution using the DISH software.^30^ This kind of analytical method uses a regularization term λ to prevent inflation due to statistical noises.^30^, ^31^ We set a small value (λ = 0.05) to prevent false negatives and consider the denoising nature of meta‐analysis. As the LD reference data of the MHC region specific for individual populations, we used that of T1DGC reference panel for European ancestries (*n* = 5225),^20^, ^32^ which was implemented in DISH. In addition, we generated the LD information of Japanese and other East Asian ancestries from our Japanese reference panel (*n* = 1120),^19^ and Pan‐Asian reference panel (*n* = 530),^32^, ^33^ respectively. Before imputation, we removed indel and multi‐allelic SNPs and aligned the strand of SNVs between the GWAS summary and the reference data according to the same criteria as SNP2HLA.^20^ For further stringent QC, we removed the SNVs of which the frequency of minor alleles in the GWAS summary were higher than 0.5 in the reference or the difference in allele frequencies between the GWAS summary and reference were higher than 0.2. We applied stringent postimputation QC filtering of the variants (minor allele frequency ≥ 0.01 and imputation score *r* ^*2*^ ≥ 0.7).

Conditional analysis of the summary statistics was performed using GCTA COJO with default parameters.^34^ The effects (β coefficients) and standard errors of imputed alleles for the analysis were calculated from the *z* scores in a manner described previously.^35^ We used the HLA reference data according to individual populations.

### 1.6 Trans‐Ethnic Meta‐Analysis for Fine‐Mapping

We meta‐analyzed the association signals of the variants shared among all the HLA reference panels used in the target studies. In trans‐ethnic meta‐analysis, considering the disparity in allele frequency of SNVs among the different populations, we removed all the palindromic SNVs to correctly align the strands. The *z* scores in the association tests of each variant were meta‐analyzed using the sample size–based meta‐analysis method, considering the heterogeneity in the distributions of β coefficients and standard errors among studies.^36^ For trans‐ethnic fine‐mapping, we performed conditional meta‐analysis by integrating the individual results of the conditional analysis. We applied a forward stepwise conditional analysis for the HLA variants. In each conditional step, we additionally included the associated variants as covariates and performed the individual analyses and meta‐analysis until no variants satisfied the significance in the meta‐analysis. We used not only a genome‐wide significance threshold of *P* = 5.0 × 10^−8^ as a strict criterion but also a study‐wide significance threshold of *P* = 3.3 × 10^−6^ based on Bonferroni correction of the total number of the HLA variants typed in the reference panels used in this study (= 0.05/15,000) to avoid missing weak but meaningful associations.

### 1.7 In Silico Prediction of Binding Affinity of *HLA‐DRB1* Alleles to α‐Synuclein

We tested the binding affinity of *HLA‐DRB1* alleles to a candidate epitope derived from α‐synuclein peptide with amino acid residue Y39 (KTKEGVLYVGSKTKE) using NetMHCIIpan 4.0 with BA option and default settings.^37^ The difference in the binding affinity (nM) between allelic groups were analyzed using the Mann‐Whitney *U* test. We targeted all four‐digit *HLA‐DRB1* alleles typed in any reference panels and supported in NetMHCIIpan 4.0.

## 2 Results

### 2.1 Overview of the Trans‐Ethnic HLA Fine‐Mapping of PD Risk

As illustrated in Figure 1, we adopted a hybrid fine‐mapping approach including both HLA genotype imputation of PD GWAS data and direct imputation of HLA variant risk from the PD GWAS summary statistics. We first performed the MHC fine‐mapping of the UKB cohort (1599 PD cases and 352,325 controls)^23^, ^24^ through HLA genotype imputation of the UKB PD GWAS data using the DEEP*HLA software.^22^ We also conducted direct imputation of PD risk statistics of the HLA variants from the PD GWAS summary statistics of Europeans (13,051 PD cases and 936,300 controls; *n*_Study_ = 4)^4^, ^6^, ^25^ and East Asians (7712 PD cases and 27,372 controls; *n*_Study_ = 2)^5^, ^26^ based on the approximation inference of *z* scores using the DISH software.^30^ Finally, we performed trans‐ethnic MHC fine‐mapping by meta‐analyzing in total seven GWAS summary statistics (22,362 PD cases and 1,315,997 controls; *n*_Study_ = 7).

### 2.2 Classes I and II HLA Genes Confer PD Susceptibility

**FIG. 2:**
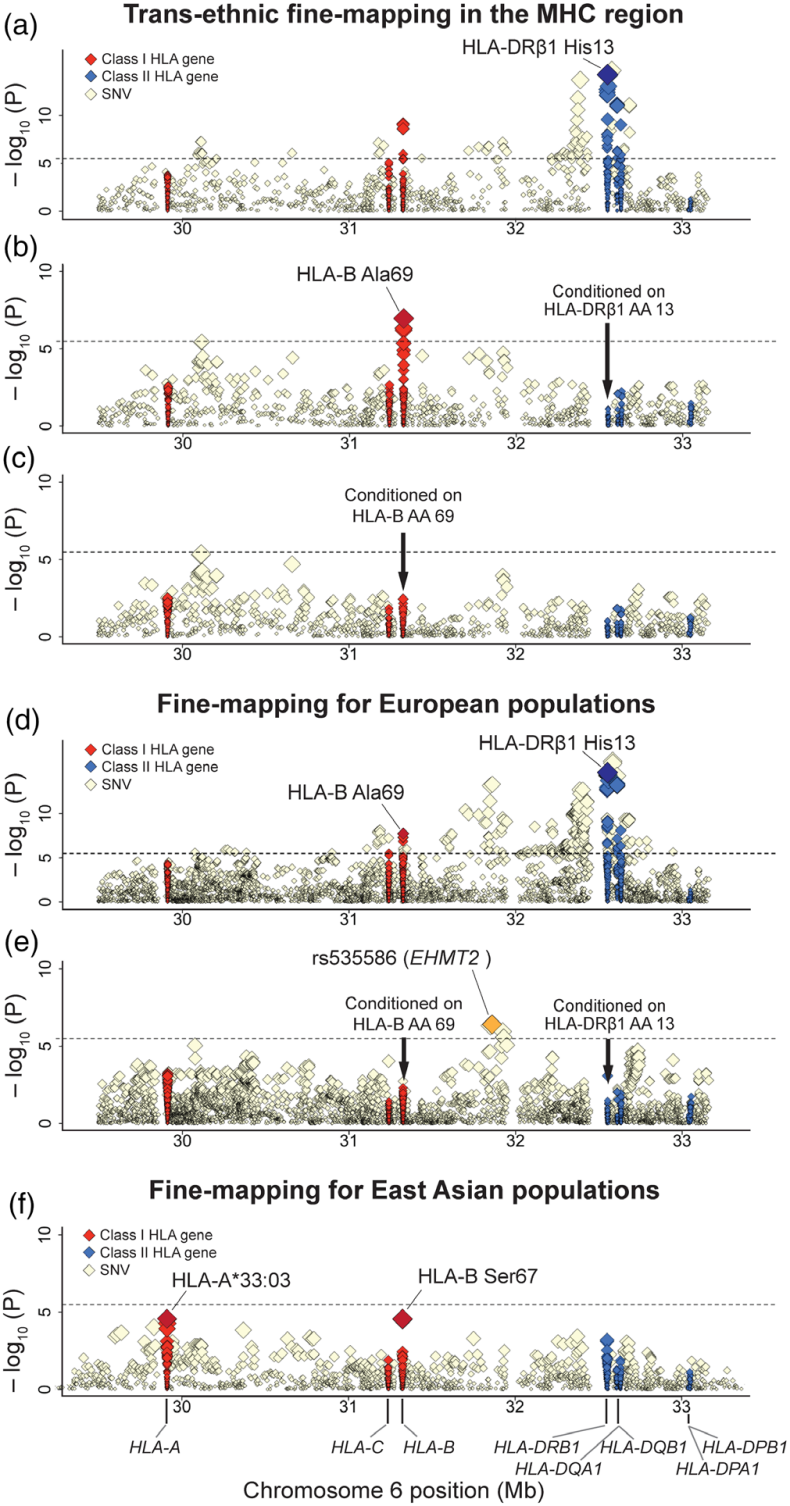
Regional association plots of the human leukocyte antigen (HLA) variants with Parkinson's disease. Diamonds represent −log_10_ (*P* values) for the tested HLA variants, including single‐nucleotide variants (SNVs), classical alleles, and amino acid (AA) polymorphisms of the HLA genes. The dashed black horizontal lines represent the study‐wide significance threshold of *P* = 3.3 × 10^−6^. The physical positions of the HLA genes on chromosome 6 are shown at the bottom. Each panel shows the association plot in the process of stepwise conditional regression analysis in the trans‐ethnic meta‐analysis: (**a**) nominal results, (**b**) results conditioned on HLA‐DRβ1 AA position 13, and (**c**) results conditioned on HLA‐DRβ1 AA position 13 and HLA‐B AA position 69. Each panel shows the association plot in the meta‐analysis in the European populations: (**d**) nominal results and (**e**) results conditioned on HLA‐DRβ1 AA position 13 and HLA‐B AA position 69. (**f**) The association plot in the meta‐analysis in the East Asian populations. [Color figure can be viewed at wileyonlinelibrary.com]

Through the trans‐ethnic HLA fine‐mapping analysis, we found the strongest signals at histidine at amino acid position 13 (His13) in HLA‐DRβ1 (*P* = 6.0 × 10^−15^; Fig. 2a). The equivalent associations were observed at the HLA‐DRB1*04 allele and its corresponding amino acid polymorphisms of asparagine or histidine at amino acid position 33 (Asn/His33) in HLA‐DRβ1 (*P* = 6.1 × 10^−15^). Since His13 in HLA‐DRβ1 is strongly tagged with HLA‐DRB1*04 (*r* ^*2*^ = 0.9995), our results were consistent with the previous studies reporting the protective effect of HLA‐DRB1*04.^10^, ^38^ To further elucidate PD risk–associated variants independently of the *HLA‐DRB1* region, we robustly conducted a conditional analysis on all the amino acid polymorphisms of HLA‐DRβ1 position 13. Then, the association signals in *HLA‐DRB1* remarkably weakened (*P* > 0.01), suggesting that the amino acid position 13 explains the majority of PD risk of *HLA‐DRB1*.

Apart from *HLA‐DRB1*, we observed the most significant independent association at alanine at amino acid position 69 (Ala69) in HLA‐B (*P* = 1.0 × 10^−7^; Fig 2b), which satisfied the study‐wide significance threshold. After conditioning on all the amino acid polymorphisms of HLA‐DRβ1 position 13 and HLA‐B position 69, no variants in the MHC region satisfied the region‐wide significance threshold (*P* > 3.3 × 10^−6^; Fig 2d). All of the risk‐associated variants obtained by trans‐ethnic meta‐analysis are summarized in Table 1 and Table S3.

**TABLE 1:** Trans‐ethnic major histocompatibility complex fine‐mapping of risk‐associated variants in Parkinson's disease

HLA variant	European populations										East Asian populations				Meta‐analysis	
	UKB		23andMe^25^		23andMe^25^		23andMe^4^		23andMe^6^		Japanese^26^		East Asian^5^			
	1599 cases and 352,325 controls		3261 cases and 29,499 controls		866 cases and 32,538 controls		6476 cases and 302,042 controls		2448 cases and 571,411 controls		988 cases and 2521 controls		779 cases and 13,227 controls		16,417 cases and 1,304,373 controls	
	OR (95% CI)^a^	*P*	OR (95% CI)^a^	*P*	OR (95% CI)^a^	*P*	OR (95% CI)^a^	*P*	OR (95% CI)^a^	*P*	OR (95% CI)^a^	P	OR (95% CI)^a^	*P*	Effect^b^	*P*
Trans‐ethnic association with PD risk (not conditioned)																
HLA‐DRβ1 amino acid position 13 (chr6: 32,552,130–32,552,132; rs9269951 [C/A], rs1136759 [C/A/G/T], and rs1136758 [T/A/C/G])^c^																
Arg (ACG, CCG, CCT)	1.11 (0.98–1.25)	0.10	1.01 (0.92–1.10)	0.82	1.04 (0.88–1.23)	0.66	1.11 (1.04–1.18)	7.5 × 10^−4^	1.11 (1.01–1.23)	0.029	0.93 (0.81–1.06)	0.28	1.11 (0.98–1.26)	0.10	Risk	9.1 × 10^−5^
Gly (ACC, CCC)	1.13 (0.97–1.31)	0.11	1.00 (0.89–1.11)	0.95	1.02 (0.84–1.26)	0.82	1.01 (0.93–1.08)	0.89	1.04 (0.92–1.17)	0.56	0.99 (0.87–1.13)	0.86	0.86 (0.77–0.97)	0.013	‐	0.58
His (ATG)	0.91 (0.84–0.98)	0.012	0.87 (0.83–0.92)	2.9 × 10^−6^	0.89 (0.80–0.99)	0.025	0.91 (0.88–0.95)	5.5 × 10^−6^	0.92 (0.87–0.98)	0.0098	1.02 (0.91–1.15)	0.75	0.81 (0.70–0.93)	0.0041	Protective	6.0 × 10^−15^
Phe (AAA)	0.92 (0.83–1.03)	0.16	1.07 (0.99–1.16)	0.11	1.15 (0.99–1.34)	0.063	1.05 (1.00–1.11)	0.063	1.03 (0.94–1.13)	0.50	1.04 (0.92–1.18)	0.52	1.16 (0.99–1.36)	0.073	Risk	0.0036
Ser (ACT, AGA, CGA)	1.02 (0.95–1.10)	0.52	1.04 (0.99–1.10)	0.11	0.98 (0.89–1.08)	0.67	1.01 (0.97–1.05)	0.64	0.99 (0.94–1.05)	0.81	1.02 (0.89–1.18)	0.74	1.17 (1.03–1.34)	0.017	Risk	0.030
Tyr (ATA)	1.05 (0.92–1.19)	0.45	1.15 (1.05–1.26)	0.0040	1.16 (0.97–1.38)	0.10	1.03 (0.97–1.10)	0.37	1.06 (0.96–1.18)	0.26	0.63 (0.23–1.73)	0.37	0.97 (0.80–1.17)	0.74	Risk	0.027
Trans‐ethnic association with PD risk (conditioned on amino acid positions of 13 in HLA‐DRβ1)																
HLA‐B amino acid position 69 (chr6: 31,324,529–31,324,531; rs41548914 [G/A/T], rs41546313 [G/A/C], and rs1131204 [C/G/T])^c^																
Ala (AGC, GGC, TGC)	1.12 (1.03–1.22)	0.012	1.09 (1.02–1.16)	0.0076	1.10 (0.97–1.24)	0.12	1.04 (1.00–1.09)	0.072	1.11 (1.04–1.19)	0.0031	1.01 (0.88–1.15)	0.91	1.17 (1.03–1.33)	0.018	Risk	1.0 × 10^−7^
Thr (AGT, GGT, TGT)	0.89 (0.82–0.98)	1.2 × 10^−2^	0.92 (0.86–0.98)	0.0089	0.91 (0.81–1.03)	0.12	0.97 (0.92–1.01)	0.11	0.90 (0.84–0.96)	0.0022	1.01 (0.90–1.14)	0.81	0.88 (0.78–0.99)	0.032	Protective	4.8 × 10^−7^

Abbreviations: 95% CI, 95% confidence interval; Ala, alanine; Arg, alginine; Gly, glycine; His, histidine; OR, odds ratio; PD, Parkinson's disease; Phe, phenylalanine; Ser, serine; Thr; threonine; Tyr, thyrosine; UKB, UK Biobank.

^a^ ORs and 95% CIs were estimated from the imputed *z* scores.

^b^ Only variants with nominally significant effects are labeled as “risk” or “protective” based on the effect direction.

^c^ Chromosome position and rs‐numbers with their alleles are shown in parenthesis. Amino acid residues only tested in the current study are shown along with their corresponding tri‐nucleotides on the genome (ie, reverse compliments of their codons) in parenthesis.

### 2.3 Ethnically Shared and Distinct PD Risk of the HLA Variants Between Europeans and East Asians

Although trans‐ethnic fine‐mapping is more suitable to detect genetic features associated with the etiology in a robust manner,^21^, ^22^ additional investigations of the responsible variants separately within each ancestry could provide insights into population‐specific genetic backgrounds of the phenotypes. We thus additionally conducted HLA fine‐mapping analyses of PD risk separately for individual populations of Europeans and East Asians.

The European meta‐analysis (14,650 PD cases and 1,288,625 controls) revealed the most significant associations at His13 in HLA‐DRβ1 (*P* = 2.3 × 10^−14^; Fig 2e) as well as its tagged SNP in strong LD (rs3104413; *P* = 1.3 × 10^−16^; *r* ^*2*^ = 0.97). When conditioning on all of the amino acid polymorphisms of HLA‐DRβ1 position 13 and HLA‐B position 69 in the same manner as the trans‐ethnic analysis, we identified additional independently associated variants that satisfied the study‐wide significance threshold at non‐HLA genes within the class III MHC region as well (rs535586 at *EHMT2*; *P* = 2.5 × 10^−7^; Fig. 2f). We were not able to evaluate the association of this SNP in trans‐ethnic fine‐mapping since rs535586 was not available in trans‐ethnic fine‐mapping because of the limited coverages of the SNVs shared among the HLA reference panels of diverse ancestries.

In the East Asian meta‐analysis (7712 PD cases and 27,372 controls), the strongest associations were observed at the class I MHC regions of *HLA‐A* and *HLA‐B*. The top signals were at HLA‐A*33:03 (risk; *P* = 2.9 × 10^−5^) and serine at amino acid position 67 (Ser67) in HLA‐B (protective; *P* = 3.2 × 10^−5^), but slightly not satisfying the study‐wide significance threshold (*P* < 3.3 × 10^−6^; Fig 2g). Since Ser67 in HLA‐B is moderately tagged with Ala69 in HLA‐B (*r* ^*2*^ = 0.30 and 0.17 in Pan‐Asian and Japanese reference panels, respectively), its signal might reflect the primary signal of Ala69. In contrast, HLA‐A*33:03 allele did not demonstrate nominal association with PD risk in the European meta‐analysis (*P* = 0.92), possibly because of the lower allele frequencies in Europeans (= 0.019) than in East Asians (= 0.10).^18^ Although the cohort of East Asian meta‐analysis showed a nominally significant protective effect of His13 in HLA‐DRβ1 (*P* = 0.0086), its association was not observed in the Japanese cohort (*P* = 0.69). These results demonstrated the ethnically shared and distinct HLA genetic architecture of PD between and within Europeans and East Asians.

### 2.4 The Risk‐Associated *HLA‐DRB1* Alleles Might Alter the Binding Affinity to α‐Synuclein

**FIG. 3:**
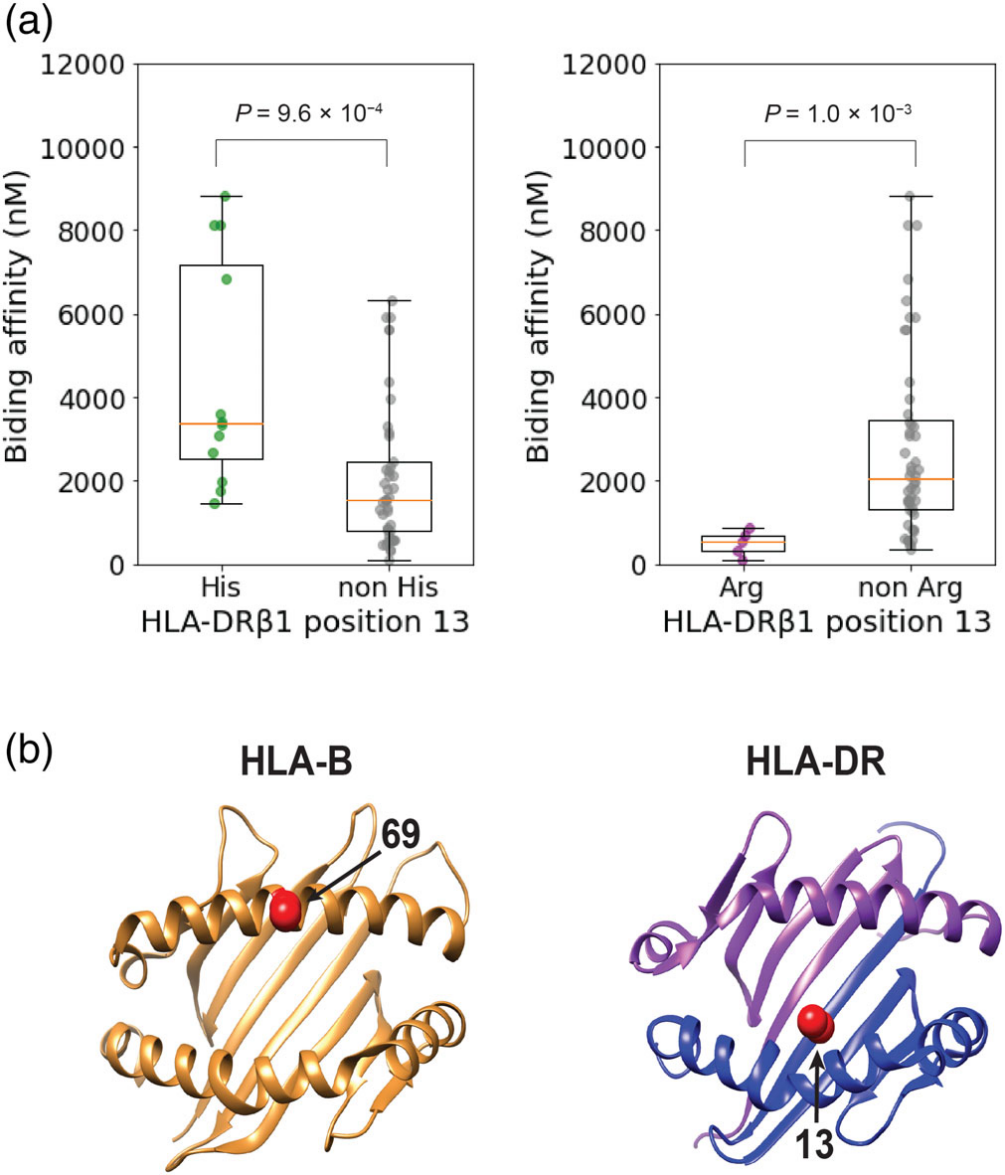
Risk‐associated amino acid polymorphisms and in silico predicted binding affinity of *HLA‐DRB1* alleles to an α‐synuclein epitope. (**a**) The results of in silico predicted binding affinity of *HLA‐DRB1* alleles to the Y39 epitope of α‐synuclein are shown in boxplots and compared between alleles with and without His (left) and Arg (right) in position 13 of HLA‐DRβ1. His13 in HLA‐DRβ1 is almost consistent with HLA‐DRB1*04, and HLA‐DRB1*15:01 has Arg13 in HLA‐DRβ1. (**b**) Three‐dimensional ribbon models of the human leukocyte antigen (HLA) proteins associated with Parkinson's disease (PD) risk. The protein structures of HLA‐B and HLA‐DR are based on Protein Data Bank entries 2BVP and 3PDO, respectively, which were displayed using UCSF Chimera version 1.14. Residues at the PD risk‐associated amino acid positions are colored red (arrows). Arg, arginine; His, histidine. [Color figure can be viewed at wileyonlinelibrary.com]

A previous study suggested that α‐synuclein–derived epitopes, especially two peptides involving amino acid positions Y39 and S129, could induce variable T cell responses according to class II HLA alleles in patients with PD.^7^ HLA‐DRB1*15:01 was highlighted because it had the strongest binding affinity to the Y39 epitope and patients with PD who responded to the Y39 epitope were more likely to have HLA‐DRB1*15:01. HLA‐DRB1*15:01 presented a suggestive association with PD risk in the current study (*P* = 4.1 × 10^−6^). Since the number of *HLA‐DRB1* alleles assayed in the previous study was limited, we comprehensively evaluated in silico–predicted binding affinities of *HLA‐DRB1* alleles to the Y39 epitope using NetMHCIIpan 4.0.^37^ As a result, HLA‐DRB1*15:01, which has arginine at amino acid position 13 (Arg13), exhibited the strongest binding affinity (97 nM) in concordance with the previous results of an in vitro assay (Table S4). Interestingly, *HLA‐DRB1* alleles with His13 and Arg13, the protective and risk allele in this position (Table 1), had significantly weaker and stronger binding affinity than others, respectively (*P* = 9.6 × 10^−4^ and 1.0 × 10^−3^, respectively; Fig 3a). These results might suggest that amino acid polymorphisms of position 13 are associated with PD risk through altering the antigen presentation of α‐synuclein–derived epitopes and successive immune responses. We did not test the S129 epitope because T cell responses induced by the S129 epitope were only observed with phosphorylation.^7^

## 3 Discussion

We conducted trans‐ethnic MHC fine‐mapping of PD risk in European and East Asian populations. We identified the most significant association signals at the protective risk of His13 of HLA‐DRβ1 along with the equivalent association in HLA‐DRB1*04. The amino acid position 13 is located in the floor of the peptide‐binding groove of the HLA‐DR molecule and is thus well positioned to directly interact with bound peptides (Fig. 3b). As noted previously, our result is also consistent with the inverse epidemiologic correlation between PD and rheumatoid arthritis, of which susceptibility risk is strongly associated with His13 in HLA‐DRβ1.^38^, ^39^, ^40^, ^41^ Whereas a recent study reported the association with “shared epitopes” of *HLA‐DRB1*,^39^ their associations satisfied neither study‐wide significance (*P* > 3.3 × 10^−6^) nor even nominal significance when conditioned on the amino acid position 13 (*P* > 0.05; Table S3). In addition, we have first identified independent suggestive associations between PD risk and the class I HLA gene of HLA‐B Ala69. HLA‐B Ala69 also composes the border of the peptide‐binding groove of the HLA‐B molecule (Fig. 2b). A previous study reported that a haplotype including HLA‐B*07:02 might confer the PD risk in Europeans.^10^ HLA‐B*07:02 has moderate LD with Ala69 in Europeans (*r* ^*2*^ = 0.35), suggesting that the previously reported HLA‐B*07:02 risk might have reflected the primary risk of Ala69 in HLA‐B.

Although the pathogenic roles of HLA molecules in the pathogenesis of PD have not been fully elucidated, recent studies suggested that α‐synuclein peptides can induce different T cell reactivity associated with HLA alleles.^7^ The same authors also reported that this reaction might occur before the onset of PD, suggesting the etiologic role of immune responses mediated by HLA.^42^ The two major regions involving Y39 and phosphorylated S129 were suggested as candidate epitopes. Our results of the in silico prediction revealed that the *HLA‐DRB1* alleles with His13 (ie, HLA‐DRB1*04 subtypes) and Arg13 have weaker and stronger binding affinity than others. This observation supports the hypothesis that His13 or HLA‐DRB1*04 exhibit a protective effect on the development of PD through the reduced binding affinity to α‐synuclein epitopes. Although the phosphorylated S129 epitope was featured in relation to the higher binding affinity of HLA‐DQB1*04:02 and HLA‐DQB1*05:01 in the previous study,^7^ the current study could not confirm the associations of both alleles with PD risk. The phosphorylated S129 epitope is also important since phosphorylated S129 residues are present in high levels in Lewy body with a significant role in the pathophysiology of PD by causing toxicity.^43^, ^44^ Therefore, a more comprehensive analysis focusing on the difference in immunologic responses to this epitope among different HLA alleles might be helpful in further elucidation of the immunologic pathophysiology of PD. The same study suggested that HLA‐A*11:01 also activated T cell responses through the presentation of the Y39 epitopes; however, HLA‐A:11:01 was not significantly associated with the risk in the current study. On the other hand, our study suggested the association of a particular amino acid position of HLA‐B with PD risk; thus, experimental validation of the epitope presentation of HLA‐B molecules to α‐synuclein peptides might contribute to the further understanding of the role of class I HLA genes in the PD etiology.

Several neurodegenerative diseases have been reported to be associated with different HLA variants.^45^, ^46^ The risk of Alzheimer's disease (AD) was observed at HLA‐DRB1*15:01, HLA‐DQA1*01:02, and HLA‐DQB1*06:02 and the haplotype composed of them (DR15),^46^ but they were not associated with PD risk even in the study‐wide significance level (*P* > 3.3 × 10^−6^). In contrast, the AD protective variants of HLA‐DQA1*03:01 and HLA‐DQB1*03:02 were significantly associated with a protective effect on PD in our study (*P* < 5.0 × 10^−8^). However, their protective effects on each of the diseases diminished when conditioned on the most risk‐associated variants, respectively.

Our results also suggested additional independent association signals at the class III MHC region where no HLA genes are located (6p21.33) in the European population. The lead variant (rs535586) was observed at a variant of *EHMT2*. Sugeno and colleagues reported that α‐synuclein in the nucleus might activate H3K9 via the EHMT2 protein, and such an epigenetic effect could affect a neural cell adhesion molecule and a synaptosomal‐associated protein, leading to the synaptic dysfunction occurring in PD.^47^ We note that rs535586 was not included in the HLA imputation panels of East Asians and thus not evaluated in the trans‐ethnic meta‐analysis. Since independent PD risk of non‐HLA variants was suggested by our study, our next step should focus on the fine‐mapping of this region using a densely typed reference panel for a larger multiethnic cohort.^21^ Since the MHC class III region is known to harbor independent risk from HLA genes on a variety of complex human traits,^48^ further fine‐mapping should provide novel insights into the genetics of PD.

Interestingly, the magnitude of the effect sizes in classes I and II HLA genes were heterogeneous between the European and East Asian populations. Of note, the effect sizes of the core risk‐associated variants (His13 in HLA‐DRβ1 and Ala69 in HLA‐B) were relatively weak in the Japanese population. In addition, HLA‐A*33:03, which is too rare to evaluate in Europeans, might have risk in East Asians. Although we cannot rule out the possibility that the observed risk heterogeneity could be attributable to a lack of statistical power because of the relatively small sample sizes in the Asian populations, the differences might reflect the population‐specific gene–gene or gene–environment interactions. To further investigate this issue, an investigation with more participants from Asian populations should be warranted.

As an additional potential limitation of the analytical methods, we applied a sample size–based meta‐analysis of the *z* score in the trans‐ethnic meta‐analysis rather than an effect size–based meta‐analysis, which made it difficult to quantify model heterogeneity among cohorts consisting of diverse ancestries. In conditional analyses on the summary statistics, the sample sizes of the Asian reference panels could not be enough to robustly maintain the reliability of COJO.^34^ These limitations derived from limited access to the individual genotype data of the PD GWAS, and the development of a secure data‐sharing scheme should be warranted.^49^ As for fine‐mapping, our approach of focusing on classical HLA alleles and amino acid polymorphisms is currently standard; however, we cannot deny the possibility that some variants have an expression quantitative trait loci (eQTL) effect on HLA genes, contributing to the etiology of PD. Because of the difficulty in quantification of HLA gene expressions,^50^ there is no reliable eQTL database especially for multiethnic populations. A future hybrid approach that also incorporates eQTL information might expedite our understanding of a functional role of variations in the MHC region on the etiology.

In summary, our study suggested that amino acid position 13 in HLA‐DRβ1 explains the majority of PD risk in *HLA‐DRB1*, and an amino acid polymorphism of *HLA‐B* might also independently confer PD risk. Considering diverse antigen‐presentation abilities among different alleles, our findings might contribute to identification of future therapeutic targets. Furthermore, independent suggestive associations of the non‐HLA variants located in the class III MHC region were observed in Europeans. Considering the potential interethnic differences in the risk‐associated genetic features, MHC fine‐mapping in a larger multiethnic cohort will provide further insight.

## Data Availability

The analysis of UK Biobank genome‐wide association study (GWAS) data was conducted via the application number 47821 (https://www.ukbiobank.ac.uk/). The Type 1 Diabetes Genetics Consortium human leukocyte antigen (HLA) reference panel can be download at a National Institute of Diabetes and Digestive and Kidney Diseases (NIDDK) central repository with a request (https://repository.niddk.nih.gov/studies/t1dgc-special/). The Japanese HLA data have been deposited at the National Bioscience Database Center Human Database (research ID hum0114). Pan‐Asian HLA reference data can be downloaded at the SNP2HLA download site (http://software.broadinstitute.org/mpg/snp2hla/). The GWAS summary statistics from 23andMe can be obtained under an agreement that protects the privacy of 23andMe research participants (https://research.23andme.com/dataset-access). The access of GWAS summary statistics of the Japanese and East Asian populations is considered upon request to the researchers of the individual original studies.

## Code Availability

We provide concise scripts to perform major histocompatibility complex fine‐mapping using summary statistics in the same manner as the current study in the GitHub repository (https://github.com/tatsuhikonaito/Trans-ethnic_MHC_fine-mapping_SS).

## Author Roles

1. Research Project: A. Conception, B. Organization, C. Execution; 2. Statistical Analysis: A. Design, B. Execution, C. Review and Critique; 3. Manuscript: A. Writing of the First Draft, B. Review and Critique.

T.N.: 1A, 1B, 2B, 3A

W.S.: 1C

K.O.: 1C

K.S.: 2C

J.H.: 1C

J.N.F.: 1C

E.‐K.T.: 1C

T.T.: 1A

Y.O.: 1A, 1B, 2A, 3B

## Financial Disclosures

This study was supported by the Japan Society for the Promotion of Science (JSPS) KAKENHI (19H01021 and 20K21834), Japan Agency for Medical Research and Development (AMED) (JP20km0405206, JP20km0405211, and JP20km0405217), the Takeda Science Foundation, and the Bioinformatics Initiative of Osaka University Graduate School of Medicine, Osaka University. T.N. was supported by JSPS KAKENHI (20J12189). J.N.F. was supported by a Singapore National Research Foundation Fellowship (NRF‐NRFF2016‐03). E.K.T. was supported by the National Medical Research Council, Singapore, Open Fund Large Collaborative Grant SPARKII (MOH‐OFLCG‐002). J.H. is an employee of Teijin Pharma Limited.

## Supporting information

**Appendix S1**: Supplementary Information

## References

[1] Poewe W, Seppi K, Tanner CM, et al. Parkinson disease. Nat Rev Dis Primers 2017;3:1–21. 10.1038/nrdp.2017.13 28332488

[2] Tan EK, Chao YX, West A, et al. Parkinson disease and the immune system — associations, mechanisms and therapeutics. Nat Rev Neurol 2020;16(6):303–318. 10.1038/s41582-020-0344-4 32332985

[3] Ibanez L, Dube U, Saef B, et al. Parkinson disease polygenic risk score is associated with Parkinson disease status and age at onset but not with alpha‐synuclein cerebrospinal fluid levels. BMC Neurol 2017;17(1):1–9. 10.1186/s12883-017-0978-z 29141588 PMC5688622

[4] Chang D, Nalls MA, Hallgrímsdóttir IB, et al. A meta‐analysis of genome‐wide association studies identifies 17 new Parkinson's disease risk loci. Nat Genet 2017;49(10):1511–1516. 10.1038/ng.3955 28892059 PMC5812477

[5] Foo JN, Tan LC, Irwan ID, et al. Genome‐wide association study of Parkinson's disease in east Asians. Hum Mol Genet 2017;26(1):226–232. 10.1093/hmg/ddw379 28011712

[6] Nalls MA, Blauwendraat C, Vallerga CL, et al. Identification of novel risk loci, causal insights, and heritable risk for Parkinson's disease: a meta‐analysis of genome‐wide association studies. Lancet Neurol 2019;18(12):1091–1102. 10.1016/s1474-4422(19)30320-5 31701892 PMC8422160

[7] Sulzer D, Alcalay RN, Garretti F, et al. T cells from patients with Parkinson's disease recognize α‐synuclein peptides. Nature 2017;546(7660):656–661. 10.1038/nature22815 28636593 PMC5626019

[8] Aliseychik MP, Andreeva TV, Rogaev EI. Immunogenetic factors of neurodegenerative diseases: the role of HLA class II. Biochemistry 2018;83(9):1104–1116. 10.1134/s0006297918090122 30472949

[9] Hamza TH, Zabetian CP, Tenesa A, et al. Common genetic variation in the HLA region is associated with late‐onset sporadic Parkinson's disease. Nat Genet 2010;42(9):781. 10.1038/ng.642 20711177 PMC2930111

[10] Wissemann WT, Hill‐Burns EM, Zabetian CP, et al. Association of parkinson disease with structural and regulatory variants in the HLA region. Am J Hum Genet 2013;93(5):984–993. 10.1016/j.ajhg.2013.10.009 24183452 PMC3824116

[11] Chuang Y‐H, Lee P‐C, Vlaar T, et al. Pooled analysis of the HLA‐DRB1 by smoking interaction in Parkinson disease. Ann Neurol 2017;82(5):655–664. 10.1002/ana.25065 28981958 PMC5798887

[12] Zhao Y, Gopalai AA, Ahmad‐Annuar A, et al. Association of HLA locus variant in parkinson's disease. Clin Genet 2013;84(5):501–504. 10.1111/cge.12024 23083294

[13] Chang K‐H, Wu Y‐R, Chen Y‐C, et al. Association of genetic variants within HLA‐DR region with Parkinson's disease in Taiwan. Neurobiol Aging 2020;87:140.e13–140.e18. 10.1016/j.neurobiolaging.2019.11.002 31818508

[14] Dendrou CA, Petersen J, Rossjohn J, Fugger L. HLA variation and disease. Nat Rev Immunol 2018;18(5):325–339. 10.1038/nri.2017.143 29292391

[15] Gourraud PA, Khankhanian P, Cereb N, et al. HLA diversity in the 1000 genomes dataset. PLoS One 2014;9(7). 10.1371/journal.pone.0097282 PMC4079705 24988075

[16] Pereyra F, Jia X, McLaren PJ, et al. The major genetic determinants of HIV‐1 control affect HLA class I peptide presentation. Science 2010;330(6010):1551–1557. 10.1126/science.1195271 21051598 PMC3235490

[17] Raychaudhuri S, Sandor C, Stahl EA, et al. Five amino acids in three HLA proteins explain most of the association between MHC and seropositive rheumatoid arthritis. Nat Genet 2012;44(3):291–296. http://www.nature.com/articles/ng.1076 22286218 10.1038/ng.1076 PMC3288335

[18] Okada Y, Momozawa Y, Ashikawa K, et al. Construction of a population‐specific HLA imputation reference panel and its application to Graves' disease risk in Japanese. Nat Genet 2015;47(7):798–802. 10.1038/ng.3310 26029868

[19] Hirata J, Hosomichi K, Sakaue S, et al. Genetic and phenotypic landscape of the major histocompatibilty complex region in the Japanese population. Nat Genet 2019;51(3):470–480. 10.1038/s41588-018-0336-0 30692682

[20] Jia X, Han B, Onengut‐Gumuscu S, et al. Imputing amino acid polymorphisms in human leukocyte antigens. PLoS One 2013;8(6);e64683. 10.1371/journal.pone.0064683 23762245 PMC3675122

[21] Luo Y, Kanai M, Choi W, et al. A high‐resolution HLA reference panel capturing global population diversity enables multi‐ethnic fine‐mapping in HIV host response. medRxiv 2020. 10.1101/2020.07.16.20155606 PMC8959399 34611364

[22] Naito T, Suzuki K, Hirata J, et al. A deep learning method for HLA imputation and trans‐ethnic MHC fine‐mapping of type 1 diabetes. Nat Commun. In press. 2021;12(1):1639. 10.1038/s41467-021-21975-x 33712626 PMC7955122

[23] Sudlow C, Gallacher J, Allen N, et al. UKbiobank: an open access resource for identifying the causes of a wide range of complex diseases of middle and old age. PLoS Med 2015;12(3):1–10. 10.1371/journal.pmed.1001779 PMC4380465 25826379

[24] Bycroft C, Freeman C, Petkova D, et al. The UKbiobank resource with deep phenotyping and genomic data. Nature 2018;562(7726):203–209. 10.1038/s41586-018-0579-z 30305743 PMC6786975

[25] Nalls MA, Pankratz N, Lill CM, et al. Large‐scale meta‐analysis of genome‐wide association data identifies six new risk loci for Parkinson's disease. Nat Genet 2014;46(9):989–993. 10.1038/ng.3043 25064009 PMC4146673

[26] Satake W, Nakabayashi Y, Mizuta I, et al. Genome‐wide association study identifies common variants at four loci as genetic risk factors for Parkinson's disease. Nat Genet 2009;41(12):1303–1307. 10.1038/ng.485 19915576

[27] Cooke GS, Hill AVS. Genetics of susceptibitlity to human infectious disease. Nat Rev Genet 2001;2(12):967–977. http://www.nature.com/articles/35103577 11733749 10.1038/35103577

[28] Price AL, Patterson NJ, Plenge RM, et al. Principal components analysis corrects for stratification in genome‐wide association studies. Nat Genet 2006;38(8):904–909. 10.1038/ng1847 16862161

[29] Foo JN, Chew EGY, Chung SJ, et al. Identification of risk loci for Parkinson disease in Asians and comparison of risk between Asians and Europeans: a genome‐wide association study. JAMA Neurol 2020;77(6):746–754. 10.1001/jamaneurol.2020.0428 32310270 PMC7171584

[30] Lim J, Bae S‐C, Kim K. Understanding HLA associations from SNP summary association statistics. Sci Rep 2019;9(1):1337. 10.1038/s41598-018-37840-9 30718717 PMC6362191

[31] Pasaniuc B, Zaitlen N, Shi H, et al. Fast and accurate imputation of summary statistics enhances evidence of functional enrichment. Bioinformatics 2014;30(20):2906–2914. 10.1093/bioinformatics/btu416 24990607 PMC4184260

[32] Han B, Diogo D, Eyre S, et al. Fine mapping seronegative and seropositive rheumatoid arthritis to shared and distinct HLA alleles by adjusting for the effects of heterogeneity. Am J Hum Genet 2014;94(4):522–532. https://dx.doi.org/10.1016%2Fj.ajhg.2014.02.013 24656864 10.1016/j.ajhg.2014.02.013 PMC3980428

[33] Pillai NE, Okada Y, Saw WY, et al. Predicting HLA alleles from high‐resolution SNP data in three Southeast Asian populations. Hum Mol Genet 2014;23(16):4443–4451. 10.1093/hmg/ddu149 24698974

[34] Yang J, Ferreira T, Morris AP, et al. Conditional and joint multiple‐SNP analysis of GWAS summary statistics identifies additional variants influencing complex traits. Nat Genet 2012;44(4):369–375. 10.1038/ng.2213 22426310 PMC3593158

[35] Zhu Z, Zhang F, Hu H, et al. Integration of summary data from GWAS and eQTL studies predicts complex trait gene targets. Nat Genet 2016;48(5):481–487. 10.1038/ng.3538 27019110

[36] de Bakker PIW, Ferreira MAR, Jia X, et al. Practical aspects of imputation‐driven meta‐analysis of genome‐wide association studies. Hum Mol Genet 2008;17(R2):122–128. 10.1093/hmg/ddn288 PMC2782358 18852200

[37] Reynisson B, Alvarez B, Paul S, et al. NetMHCpan‐4.1 and NetMHCIIpan‐4.0: improved predictions of MHC antigen presentation by concurrent motif deconvolution and integration of MS MHC eluted ligand data. Nucleic Acids Res 2020;48(W1):W449–W454. 10.1093/nar/gkaa379 32406916 PMC7319546

[38] Ahmed I, Tamouza R, Delord M, et al. Association between Parkinson's disease and the HLA‐DRB1 locus. Mov Disord 2012;27(9):1104–1110. 10.1002/mds.25035 22807207

[39] Hollenbach JA, Norman PJ, Creary LE, et al. A specific amino acid motif of HLA‐DRB1 mediates risk and interacts with smoking history in Parkinson's disease. Proc Natl Acad Sci U S A 2019;116(15):7419–7424. 10.1073/pnas.1821778116 30910980 PMC6462083

[40] Okada Y, Kim K, Han B, et al. Risk for ACPA‐positive rheumatoid arthritis is driven by shared HLA amino acid polymorphisms in Asian and European populations. Hum Mol Genet 2014;23(25):6916–6926. 10.1093/hmg/ddu387 25070946 PMC4245039

[41] Sung Y, Liu F, Lin C‐C, et al. Reduced risk of Parkinson disease in patients with rheumatoid arthritis. Mayo Clin Proc 2016;91(10):1346–1353. 10.1016/j.mayocp.2016.06.023 27712633

[42] Lindestam Arlehamn CS, Dhanwani R, Pham J, et al. α‐synuclein‐specific T cell reactivity is associated with preclinical and early Parkinson's disease. Nat Commun 2020;11(1):1875. 10.1038/s41467-020-15626-w 32313102 PMC7171193

[43] Fujiwara H, Hasegawa M, Dohmae N, et al. α‐Synuclein is phosphorylated in synucleinopathy lesions. Nat Cell Biol 2002;4(2):160–164. 10.1038/ncb748 11813001

[44] Ma MR, Hu ZW, Zhao YF, et al. Phosphorylation induces distinct alpha‐synuclein strain formation. Sci Rep 2016;6:1–11. 10.1038/srep37130 27853185 PMC5112567

[45] Zhang M, Ferrari R, Tartaglia MC, et al. A C6orf10/LOC101929163 locus is associated with age of onset in C9orf72 carriers. Brain 2018;141(10):2895–2907. 10.1093/brain/awy238 30252044 PMC6158742

[46] Kunkle BW, Grenier‐Boley B, Sims R, et al. Genetic meta‐analysis of diagnosed Alzheimer's disease identifies new risk loci and implicates Aβ, tau, immunity and lipid processing. Nat Genet 2019;51(3):414–430. 10.1038/s41588-019-0358-2 30820047 PMC6463297

[47] Sugeno N, Jäckel S, Voigt A, et al. α‐Synuclein enhances histone H3 lysine‐9 dimethylation and H3K9me2‐dependent transcriptional responses. Sci Rep 2016;6:1–11. 10.1038/srep36328 27808254 PMC5093762

[48] Kamitaki N, Sekar A, Handsaker RE, et al. Complement genes contribute sex‐biased vulnerability in diverse disorders. Nature 2020;582(7813):577–581. 10.1038/s41586-020-2277-x 32499649 PMC7319891

[49] Bonomi L, Huang Y, Ohno‐Machado L. Privacy challenges and research opportunities for genomic data sharing. Nat Genet 2020;52(7):646–654. 10.1038/s41588-020-0651-0 32601475 PMC7761157

[50] Gutierrez‐Arcelus M, Baglaenko Y, Arora J, et al. Allele‐specific expression changes dynamically during T cell activation in HLA and other autoimmune loci. Nat Genet 2020;52(3):247–253. 10.1038/s41588-020-0579-4 32066938 PMC7135372

